# High Curl Pattern Hair and Scalp Care Considerations to Mitigate Seborrheic Dermatitis

**DOI:** 10.1111/jocd.70842

**Published:** 2026-04-17

**Authors:** Valerie Callender, Cheryl Burgess, Valerie M. Harvey, Candrice Heath, Hope Mitchell, Heather Woolery‐Lloyd, Anneke Andriessen, Amy McMichael

**Affiliations:** ^1^ Department of Dermatology, College of Medicine Howard University, Callender Dermatology & Cosmetic Center Glenn Dale Maryland USA; ^2^ Department of Dermatology Georgetown University and the George Washington University, Center for Dermatology and Dermatologic Surgery Washington DC USA; ^3^ Board‐Certified Dermatologist, Newport News Virginia USA; ^4^ Department of Dermatology Howard University Washington DC USA; ^5^ Mitchell Dermatology Perrysburg Ohio USA; ^6^ Skin of the Color Division University of Miami Department of Dermatology Miami Florida USA; ^7^ RBC Consultants, Anneke Andriessen & co BV Malden the Netherlands; ^8^ Board‐Certified Dermatologist, Department of Dermatology Wake Forest School of Medicine Winston‐Salem North Carolina USA

## Abstract

**Background:**

High‐curl‐pattern hair has unique structural characteristics that increase its susceptibility to breakage and to seborrheic dermatitis (SD).

**Aims:**

The paper discusses the challenges in effectively treating patients with high‐curl‐pattern hair and SD, and the role of ceramide‐containing products in improving the condition.

**Methods:**

Following a systematic literature review, a panel of dermatologists developed five consensus statements on the relationship between SD and scalp barrier health in hair with a high curl pattern.

**Results:**

Certain hair care practices common among individuals with high‐curl‐pattern hair may contribute to the development or exacerbation of SD, leading to considerable psychological distress. These statements reflect expert opinion and highlight key treatment challenges in this population.

**Conclusion:**

Further research is needed to better understand racial and ethnic variations in SD and to support individualized, culturally sensitive approaches to its treatment and management.

## 1 Introduction

Hair and scalp disorders are consistently cited as one of the leading dermatological problems in individuals with high‐curl‐pattern hair (HCPH) [1]. HCPH is characterized by a tightly coiled structure and tends to be more prone to dryness, breakage, and damage, mainly due to the poor penetration of natural oils on the scalp [2].

Various techniques (e.g., chemical relaxers, chemical straighteners, and heat) have been used to style HCPH. Many of these hairstyles are associated with hair and scalp disorders, which can lead to significant psychological distress, along with feelings of uneasiness, frustration, and poor body image [3]. Both seborrheic dermatitis (SD) and traction alopecia are associated with hairstyles and hair products often used in HCPH. SD and dandruff are continuous spectrums of the same condition, with varying degrees of severity; scalp SD is known as dandruff [4].

This consensus paper reviews the literature on the relationship between dandruff and scalp barrier health in individuals with HCPH, including the underlying mechanisms and potential impact on overall scalp health.

## 2 Methods

**FIGURE 1:**
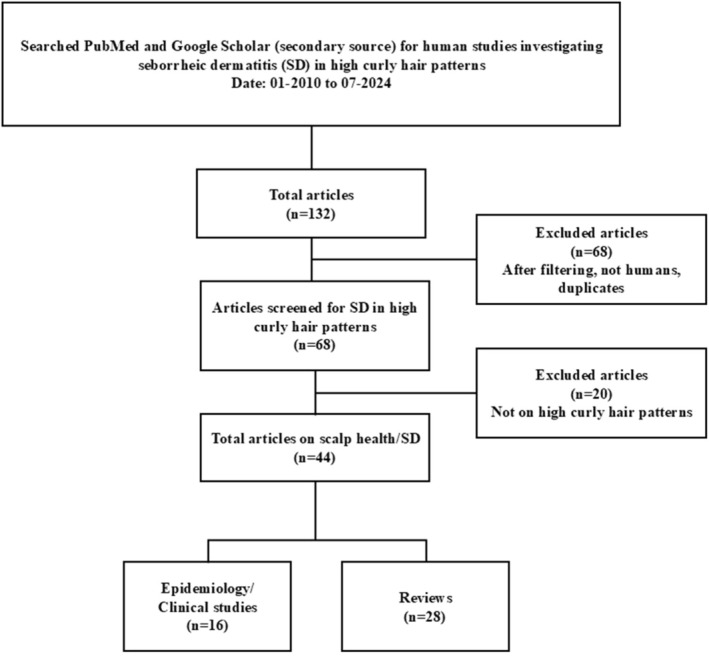
Human studies investigating seborrheic dermatitis in high curl hair patterns [01–2010 to 07–2024].

**FIGURE 2:**
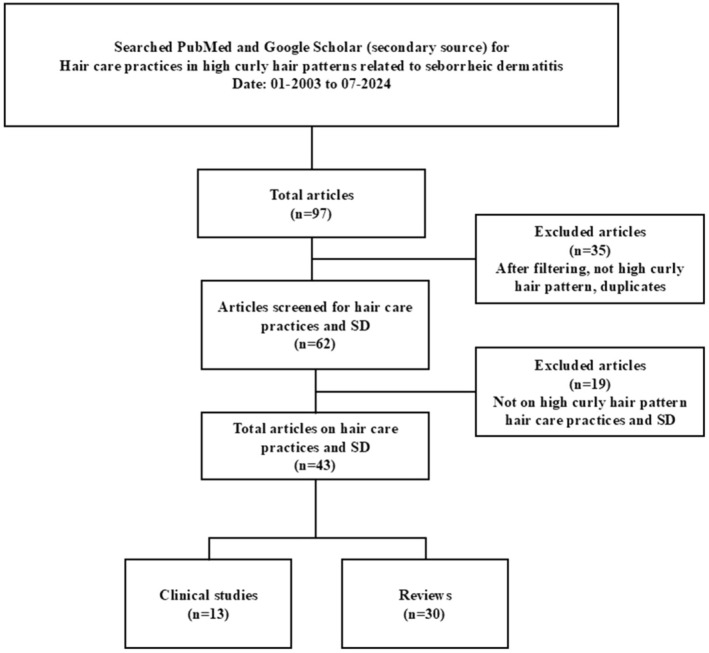
Hair care practices in high curl pattern hair related to seborrheic dermatitis [01–2003 to 07–2024].

This project started with a systematic literature review, followed by the development of consensus statements, expert discussion, and refinement of the statements. A systematic literature review was conducted on the relationship between seborrheic dermatitis and scalp barrier health in patients with HCPH, and the results were used to inform the development of draft statements. Searches for English‐language literature were conducted on December 8, 2024, in PubMed, with Google Scholar as a secondary source. The literature review objectives, search terms, and parameters are summarized in Table 1. Titles and abstracts were reviewed first, followed by the full articles. The searches for human studies investigating SD in HCPH yielded 44 papers (Figure 1), whereas the searches for hair care practices in HCPH related to SD yielded 43 papers (Figure 2). The studies that met the inclusion criteria informed the development of draft statements, which were subsequently evaluated in a face‐to‐face meeting. A panel of advisors discussed the statements during a workshop. Five consensus statements were selected based on expert opinions and discussions regarding the relationship between dandruff and skin barrier health, as well as treatment challenges for patients with HCPH.

**TABLE 1:** Literature Search Objectives and Parameters.

Scope: Human studies investigating seborrheic dermatitis in HCPH, and hair care practices in HCPH related to seborrheic dermatitis
Search Terms:
Search Set 1: Human studies investigating scalp health and seborrheic dermatitis (SD) in high curly hair patterns AND Prevalence OR Incidence OR burden of disease OR quality of life OR Psychology of hair and self‐perception OR etiopathogenesis OR scalp barrier properties OR risk factors OR diagnostics OR clinical characteristics OR management
Search Set 2: Hair care practices in HCPH related to seborrheic dermatitis AND Hair care practices Or products OR relaxers OR oils OR shampoo OR conditioner OR hair traction OR braiding OR Afro‐Ethnic hairstyling OR extensions
Included: Randomized controlled trials, observational, cohort, and interventional studies, reviews, systematic reviews, guidelines, consensus, and pathways published in English from January 2010 to July 2024 for Search set 1, and January 2003 to July 2024 for Search set 2
Excluded: Publications outside the date range, preclinical studies not addressing seborrheic dermatitis in HCPH, and publications in languages other than English

## 3 Results

Statement 1 *Seborrheic dermatitis (SD) is a common multifactorial chronic inflammatory skin condition with variable presentation. In patients with skin of color, erythema is less common as a symptom of SD, whereas SD‐associated hypopigmentation and hyperpigmentation are more common.*

SD is a chronic, benign inflammatory condition that affects areas of high sebum production [5]. SD affects 3% to 12% of the general population, with higher incidence among specific populations, such as African Americans (6.5%) and West Africans (2.9%–6%) [5].

In adults, SD is typically characterized as greasy scaly plaques on areas of the body highly populated with sebaceous glands: scalp, glabella, eyebrows, nasolabial folds, paranasal skin, ears, cheeks, upper chest, back, and skin flexures [5].

In individuals with HCPH, dandruff is a common manifestation of SD on the scalp. A cross‐sectional, multicenter study in Dakar, Senegal, that recorded 111 cases of SD in a population of 6330 patients, found that scaling was present in 88% (*n* = 98) of patients [6]. Patients with scalp involvement experienced small, non‐adherent scales forming a dandruff‐like condition, or a shiny, scaly helmet enveloping the hair. Erythema, which is more difficult to see on darker skin, is a less common symptom of SD in SOC [5, 6]. In the study above, erythema was only noted in 30.6% of patients [6]. On darker skin types, hypopigmentation often replaces erythema, but generally improves with treatment [6]. Pediatric patients with skin of color, who often have HCPH, also experience a unique SD presentation, with symptoms of erythema, flaking, and hypopigmentation of the affected areas and folds of skin, rather than the classic “cradle cap” appearance of SD [5].

Petaloid lesions (pink or hypopigmented lesions with coalescing rings and little to no scale) are also more common in SOC populations with SD. If alopecia is present, it requires aggressive treatment of the underlying SD to minimize further hair loss [7].

Statement 2 *The scalp of individuals with high curl pattern hair is perceived to be dry despite more sebum production due to the twisted structure, which inhibits sebum transport down the hair shaft.*

In individuals with HCPH, the curled configuration of the shaft impairs the distribution of natural oils, contributing to dryness of the scalp and hair [8]. Poor penetration of the natural oils on the scalp and the very curly hair texture may also contribute to dry hair in this population, despite increased sebum production. HCPH is drier, more brittle, and more susceptible to chemical and physical damage [3, 8]. In addition to intrinsic factors (Table 2), harsh cleansing and product buildup can also contribute to scalp and hair dryness, highlighting the importance of appropriate hair care in those with HCPH [9, 10, 11, 12, 13].

**TABLE 2:** Intrinsic factors contributing to scalp and hair dryness in individuals with high curl pattern hair [9, 10, 11, 12, 13].

Article title	Key findings	References
Ethnicity and stratum corneum ceramides	‐ In a study investigating differences in stratum corneum lipids among different ethnicities, the ceramide/cholesterol ratio was lowest in Africans, Caucasians had intermediate values, and highest in Asians	Jungersted JM, et al. *Br J Dermatol*. 2010;163 (6):1169–1173.
Structural and functional differences in barrier properties of African American, Caucasian and East Asian skin	‐ African American skin was shown to have low ceramide levels. ‐ TEWL has been found to be greater in Black skin compared to white skin	Muizzuddin N, et al. *J Dermatol Sci*. 2010;59 (2):123–128.
In vivo and in vitro approaches in understanding the differences between Caucasian and African skin types: specific involvement of the papillary dermis	‐ The dermis of Black individuals is thicker and more compact than that of white skin	Girardeau‐Hubert S, et al. *Int J Dermatol*. 2012;51 Suppl 1:1–4.
The Ethnic Differences of the Damage of Hair and Integral Hair Lipid after Ultra Violet Radiation	‐ Black hair breaks more easily than Caucasian hair due to the lesser tensile strength and lower moisture content ‐ African hair showed the most hair surface damage following UV irradiation	Ji JH, et al. *Ann Dermatol*. 2013;25 (1):54–60.

It is important for clinicians to differentiate between scalp dryness and SD as both conditions present with flaking but require different management. Although clinicians rely on objective signs to differentiate SD from scalp dryness, patients may present with a primary concern of “dry scalp” or “dandruff.” Since scalp inflammation may be less visible with HCPH, subjective complaints such as flaking or itching may be attributed solely to dryness. However, the patient may have underlying SD, especially when symptoms are persistent, recurrent, or resistant to moisturizing treatments. Objective assessments that clinicians can perform during a clinic visit to differentiate between scalp dryness and SD include [14, 15]:

- Expert‐assessed visual flake grading (i.e., flaking judged on a 0–10‐point scale for each of eight regions of the scalp, yielding a total adherent scalp flaking score [ASFS] ranging from 0 to 80).
- Trichoscopic examination (dermoscopic features of SD include greasy, yellow‐white scales that adhere to the scalp, arborizing vessels, atypical red vessels, and featureless areas, and normal hair shaft appearance).
- Assessment of biomarkers representing inflammation, oxidative stress, and barrier function via scalp skin tape strips.

When evaluating patients who report scalp dryness or present with scalp scaling, a thorough differential diagnosis should include SD, atopic dermatitis, contact dermatitis, psoriasis, rosacea, tinea capitis, and Langerhans cell histiocytosis [5].

Educating patients on the underlying cause of their symptoms is essential for setting appropriate expectations and improving adherence to therapy. Whereas scalp dryness may improve with moisturization, SD involves inflammation and the proliferation of the yeast genus *Malassezia*, thus requiring specialized treatment [5].

Statement 3 *Various hair care practices, including the use of straighteners and relaxers, predispose individuals with high curl patterns to hair breakage.*

HCPH is more vulnerable than other types of hair due to its high curvature and distinct follicular morphology. It exhibits an elliptical cross‐section and retro‐curvature at the bulb, resulting in an asymmetrical, S‐shaped follicle. These unique features make HCPH less resistant to mechanical extension and more prone to premature failure and breakage. The innately more fragile hair shaft, combined with certain hair care practices (Table 3), can lead to hair breakage and a dry, inflamed scalp [2, 3].

**TABLE 3:** High curl pattern hair and hair care practices.

Condition/subject	Article title	References
SD	Unmet needs for patients with seborrheic dermatitis.	Jackson JM, et al. J Am Acad Dermatol. 2024;90 (3):597–604.
SD	Dermatological conditions in skin of color‐approach to treating seborrheic dermatitis in skin of color.	Sangh AM. J Clin Aesthet Dermatol. 2024;17 (5–6 Suppl 1):S20–S23.
SD hair oils	Hair oils may worsen seborrheic dermatitis in Black patients.	Mayo T et al. Skin Appendage Disord. 2023;9 (2):151–152.
Alopecia due to hair care	Knowledge of traction alopecia and hair care practices among adolescents in Keffi, North‐Central Nigeria.	Okoro OE rt. al. Skin Appendage Disord 2022;8:129–35.22.
Hairstyling risks	Afro‐Ethnic hairstyling trends, risks, and recommendations.	Asbec k S et al. Cosmetics 2022;9:17.
Hair products	Hair cosmetics: An overview. Int J Trichology 2015;7:2–15.41.	Gavazzoni Dias MF. Int J Trichology 2015;7:2–15.41.
SD hair oils	African oils in dermatology.	Ayanlowo O et al. Dermatol Ther 2022;35:e14968.
Relaxers	Why some black women are going back to relaxers	Biakolo K et al. Allure. com 2021.
Braiding	Quantifying the impact of braiding and combing on the integrity of natural African hair.	Molamodi K, et al. Int J Cosmet Sci 2021;43:321–31.
Hair extensions	Synthetic hair extensions causing irritant contact dermatitis in patients with a history of atopy: A report of 10 cases.	Dlova NC et al. Contact Dermatitis 2021;85:141–5.
Hair care	Healthy hair care practices: caring for the African type hair.	Ekpudu *V. Niger* J Dermatol 2021;11.
Hair straightening	Straight to the point: What do we know so far on hair straightening?	Barreto T et al. Skin Appendage Disord 2021;7:265–71.
SD SOC	The management of seborrheic dermatitis 2020: An update.	Widaty S et al. J Gen Proced Dermatol Venereol Indones. 2020;5 (1):19–27.
Hair care SOC	How to Select a Good Shampoo and Conditioner.	Dias MF, et al. Hair and Scalp Treatments: APractical Guide. Cham: Springer International Publishing; 2020. p. 253–64.
SD SOC	Seborrheic dermatitis.	Suh DH et al. Fitzpatrick's dermatology. 9th ed. Vol 1. New York: McGraw‐Hill Education; 2019:428–36.
Alopecia	Clinical recognition and management of alopecia in women of color.	Raffi J, Int J Womens Dermatol. 2019;5 (5):314–319.
Risk factors	Skin lighteners and hair relaxers as risk factors for breast cancer: results from the Ghana breast health study.	Brinton LA et al. Carcinogenesis 2018;39:571–9.
SD SOC	Scalp and hair disorders at the dermatology outpatient clinic of a tertiary hospital.	Ayanlowo O. P H Med J 2017;11:127.
SD SOC	Hair and scalp disorders in adult and pediatric patients with skin of color.	Taylor SC, et al. Cutis. 2017;100 (1):31–35.
Hair care SOC	Everyday hair discourses of African black women. Qual Sociol Rev. 2017;13:158–72.	Majali Z, et al. Qual Sociol Rev. 2017;13:158–72.
Hair care SOC	My hair or health: Investigating the impact of hair care and maintenance on the health of African American women.	Shern SN. University of Cincinnati; 2017. etd.ohiolink.
Hair and disorders	Updates in the understanding and treatments of skin & hair disorders in women of color.	Lawson CN et al. Int J Womens Dermatol 2017;3:S21‐37.

Abbreviations: SD = seborrheic dermatitis, SOC = skin of color.

Various hair care practices (e.g., relaxers and straighteners) that individuals with HCPH sometimes use can lead to scalp irritation and, eventually, hair breakage (Table 4) [16, 17, 18, 19]. In a cross‐sectional survey of 727 Nigerian women, those with relaxed hair experienced significantly more scalp flaking, hair breakage, and hair loss than those with natural hair [3]. Hair extensions are also associated with higher rates of SD. In a study of African American girls aged 1 to 15 years old, a significantly higher risk of SD was associated with the use of hair extensions: adjusted odds ratio = 2.37 (95% CI 1.03–5.47, *p* = 0.04) [20].

**TABLE 4:** Impact of chemical relaxers and straighteners on hair [16, 17, 18, 19].

Article title	Key findings	References
‘Relaxers’ damage hair: Evidence from amino acid analysis	Chemical hair relaxers damage hair and can cause hair loss, with the cicatricial alopecia (CCCA) type of hair loss being common among African American women	Khumalo NP, et al. *Journal of the American Academy of Dermatology*. 2010;62 (3):402–408.
Medical and Environmental Risk Factors for the Development of Central Centrifugal Cicatricial Alopecia: A Population Study	Most forms of hair grooming methods used by African Americans, including braids, weaves, and chemical relaxers, have been linked to the development of CCCA.	Kyei A, et al. *Archives of Dermatology*. 2011;147 (8):909–914.
Clinical and anthropological perspectives on chemical relaxing of afro‐textured hair	Relaxers can cause scalp irritation, burning, and hair loss.	Aryiku SA, et al. *Journal of the European Academy of Dermatology and Venereology*. 2015;29 (9):1689–1695.
Effects of chemical straighteners on the hair shaft and scalp	Application of chemical straighteners to the hair can cause structural damage, such as porosity, and a reduction in hair strength.	de Paula JNH, et al. *An Bras Dermatol*. 2022;97:193–203.

Abbreviation: CCCA = central centrifugal cicatricial alopecia.

Both straighteners and relaxers alter hair chemistry. HCPH has a higher density of disulfide bonds formed by cysteine residues, which are key to its tightly coiled texture. A greater number of cysteine residues leads to more disulfide bonds, which may contribute to HCPH's distinctive shape and structure [2].

Chemical relaxers produce permanent hair straightening by rearranging disulfide bonds within the shaft [9]. Thermal straightening, known as hot combing or pressing, also interferes with hair chemistry by temporarily rearranging hydrogen bonds within the hair shafts [9].

The application of hair care products incompatible with HCPH can also increase hair breakage and have detrimental effects on the scalp [2]. The use of harsh shampoos and poor scalp moisturizing can lead to excessive dryness and predispose individuals to hair loss [3].

Additional factors that predispose HCPH to breakage include changes in temperature, pollution, and environmental stressors. The increased sensitivity of HCPH to these extrinsic factors can be attributed to its unique structural variations, which make it more prone to mechanical stress‐induced breakage [2].

Although various hair care practices predispose individuals with HCPH to breakage, there is an emerging trend of individuals transitioning from chemically and thermally treated hairstyles to natural (non‐chemically treated or relaxed) hairstyles [3]. A study conducted among women of African descent in London, UK, revealed that 52.6% had natural (untreated) hair than relaxed hair [1]. This shift is also gaining traction in Western countries.

Furthermore, age plays a role in shaping hair care behaviors. In their paper on the subjective experience of curly hair manageability, Daniels et al. (2024) found a significantly positive relationship between age and perceiving hair care as self‐care (*r*(506) = 0.112, *p* = 0.012). A significant positive correlation between curl type and considering investing time into hair care (*r*(506) = 0.176, *p* < 0.001) and being knowledgeable about hair being important (*r*(506) = 0.198, *p* < 0.001) was also observed [21].

Statement 4 *Hair care practices in individuals with high curl patterns depend on hairstyle choice. During SD treatment, clinicians should discuss with patients considerations for shampoo use, hair‐washing frequency, and hairstyles.*

Hair‐styling choices often determine hair‐washing frequency among individuals with HCPH. In general, Black women tend to shampoo less frequently than other ethnicities, not only due to lower levels of sebum on the hair shaft—with dryness and breakage if shampooed too frequently—but also because certain hairstyles (e.g., braiding and weaving) are time‐consuming and costly. Therefore, hair is washed less frequently to keep these hairstyles in place longer [9]. One study found that Black women with natural hairstyles washed their hair on average every 14 days, whereas those with braid or weave styles washed their hair every 18 to 32 days [1, 3]. However, SD of the scalp can be exacerbated by infrequent shampooing and can be more challenging to treat in individuals who wash their hair less frequently [5].

During SD treatment, clinicians should discuss shampoo use and hair washing frequency with patients. Shampooing at least once a week or every other week is generally recommended [22]. However, it is important to recognize that SOC patients may have cultural differences in hair care practices, and these should be considered when discussing SD management in these populations.

Since HCPH tends to be more prone to dryness, breakage, and damage, it requires more robust moisturizing and nourishing ingredients to stay hydrated and healthy [2]. To remove scaling buildup without stripping the scalp or hair, patients should be encouraged to use moisturizing and conditioning shampoos that are more gentle on the hair and scalp than traditional shampoos [3]. Patients should be educated to apply shampoo directly to the scalp versus the hair to minimize dryness [22]. Post‐shampoo conditioning and daily leave‐in moisturizers are also important to hydrate the hair.

Ceramides play a key role in maintaining scalp barrier function and should be considered when choosing shampoos for SD. As a major lipid component of the scalp's stratum corneum, ceramides are found in the lipid lamellae that surround corneocytes and are a crucial component of the skin barrier function [23]. Biochemical analysis of dandruff‐affected scalp revealed that dandruff was associated with a dramatic decrease in free lipid levels, with significant decreases in ceramides, fatty acids, and cholesterol [23]. Specifically, a significant reduction in ceramides NP and AP was observed in dandruff scalps [24]. Barrier disruption due to reduced ceramide levels is associated with increased transepidermal water loss and may facilitate *Malassezia* overgrowth, contributing to the inflammation and flaking characteristic of SD [23]. Ceramide‐containing hair care products could be among the characteristics to consider when patients with SD are selecting hair care. Research indicates that ceramides are relevant to barrier function, reducing epidermal water loss and accelerating barrier repair,25 suggesting that ceramide‐containing shampoos should be among the characteristics to consider when patients with SD select hair care products.

Patients with HCPH should be counseled not to tolerate pain during hairstyling and to avoid frequent braiding or weaving [3]. In addition, clinicians can recommend that most women of color shampoo weekly or every two weeks, and men of color shampoo more frequently (2–3 times per week) with moisturizing shampoos. Although patients may use over the counter pomades and oils to mask dry flakes or because they believe flaking represents a dry scalp, these products often worsen SD and should be avoided [5].

Statement 5 *Recommendations for the individual patient on additional measures should be practical and may include:*

- *Using an antifungal shampoo to treat scalp SD.*
- *Use prescription ointments, oils, lotions, creams, or leave‐in foams for patients whose haircare does not involve daily washing.*

Patients who fail to control their symptoms with cleansing and moisturizing alone may benefit from adopting additional treatment measures [25]. Escalating therapy in SD is usually considered when OTC measures fail to control symptoms or when disease severity interferes with quality of life. Traditional SD treatment includes topical corticosteroids or antifungals, such as ketoconazole, selenium sulfide, and zinc pyrithione‐containing shampoos/creams/lotions, used several times per week [5, 7].

Individuals with HCPH may find certain shampoos and solution‐based topicals too drying or irritating when treating SD [5]. Prescription‐strength ketoconazole shampoo is less commonly recommended for Black women and may be more appropriate for men, as hair fragility is less of a concern for men. Black women are advised to use anti‐dandruff shampoos containing zinc pyrithione, selenium sulfide, or tar carefully to avoid hair shaft damage and dryness. Patients should be informed that prolonged contact time is not needed for medicated shampoos to be effective. The shampoo should be applied directly to the scalp rather than the hair shafts to minimize dryness [5, 22].

Furthermore, the choice of treatment vehicle is important. A study by Chappell et al. [26]. found that Caucasian patients preferred antifungal foams, gels, and sprays, whereas Black patients preferred ointment or oil preparations. Using less drying formulations may improve adherence and treatment success in individuals with HCPH and help prevent hair damage and breakage [5].

## 4 Limitations

There is a paucity of data on SD and scalp barrier health in individuals with HCPH. Furthermore, cultural practices, hair‐grooming behaviors, and access to care vary widely and were not consistently accounted for in the literature, potentially affecting the interpretation of findings and subsequent treatment recommendations.

## 5 Conclusions

HCPH has distinct properties that contribute to increased susceptibility to breakage and SD. Optimizing hair and scalp care with appropriate recommendations can improve scalp and hair health. Communication about appropriate hair practices must consider patients' hair type, personal preferences, hair products, and hair grooming practices. As research continues to evolve, a deeper understanding of HCPH's unique vulnerabilities will inform more effective and inclusive dermatologic care.

## Author Contributions

All authors (V.C., C.B., V.M.H., C.H., H.M., H.W.‐L., A.A., A.M.) participated in developing the supplement and reviewed the manuscript, agreeing with its content and publication.

## Funding

This work was supported by CeraVe International‐L’Oréal Groupe.

## Ethics Statement

The authors confirm that the ethical policies of the journal, as noted on the journal's author guidelines page, have been adhered to. No ethical approval was required as this is a review article with no original research data.

## Conflicts of Interest Statement

The authors (V.C., C.B., V.M.H., C.H., H.M., H.W.‐L., A.A., A.M.) disclose receipt of an unrestricted educational grant from CeraVe International—L'Oréal Groupe; they also received consultancy fees for their work on this project. V.C. disclosed a conflicts of interest with L'Oreal as a researcher, consultant, and speaker. All authors have no other conflicts of interest with the content of this manuscript.

## Data Availability

The data that support the findings of this study are available from the corresponding author upon reasonable request.
